# Neurological Disorders Associated With COVID-19 Hospital Admissions: Experience of a Single Tertiary Healthcare Center

**DOI:** 10.3389/fneur.2021.640017

**Published:** 2021-02-19

**Authors:** Permesh Singh Dhillon, Robert A. Dineen, Haley Morris, Radu Tanasescu, Esmaeil Nikfekr, Jonathan Evans, Cris S. Constantinescu, Akram A. Hosseini

**Affiliations:** ^1^Division of Clinical Neuroscience, School of Medicine, University of Nottingham, Nottingham, United Kingdom; ^2^Department of Interventional Neuroradiology, Nottingham University Hospitals NHS Trust, Nottingham, United Kingdom; ^3^NIHR Nottingham Biomedical Research Centre, Nottingham, United Kingdom; ^4^Department of Neurology, Nottingham University Hospitals NHS Trust, Nottingham, United Kingdom

**Keywords:** COVID-19, neurology, delirium, stroke, encephalitis

## Abstract

**Background:** Early reports have detailed a range of neurological symptoms in patients with the SARS-CoV-2 infection. However, there is a lack of detailed description and incidence of the neurological disorders amongst hospitalized COVID-19 patients. We describe a range of neurological disorders (other than non-specific neurological symptoms), including their clinical, radiological, and laboratory findings, encountered in our cohort of COVID-19 patients admitted to a large tertiary institution.

**Methods:** We reviewed our prospectively collated database of all adult Neurology referrals, Neurology and Stroke admissions and Neurological multi-disciplinary team meetings for all hospitalized patients with suspected or proven COVID-19 from 17 March 2020 to 31 August 2020.

**Results:** Twenty-nine of 1,243 COVID-19 inpatients (2.3%) presented with COVID-19-related neurological disorders. The mean age was 68.9 ± 13.5(SD) years, age range of 34–97 years, and there were 16 males. Twenty two patients had confirmed, five were probable and two had suspected COVID-19 infection according to the WHO case classification. Eight patients (27%) required critical care admission. Neurological symptoms at presentation included acute confusion and delirium, seizures, and new focal neurological deficits. Based on the pre-defined neurological phenotype, COVID-19 patients were grouped into four main categories. Sixteen patients had cerebrovascular events (13 with acute ischemic stroke and three had hemorrhagic features), seven patients were found to have inflammatory, non-inflammatory and autoimmune encephalopathy (including two with known Multiple Sclerosis), whilst disorders of movement and peripheral nervous system were diagnosed in three patients each.

**Conclusion:** Although the exact prevalence and etiology remain unclear, new onset of neurological disorders, in addition to anosmia, is non-sporadic during the acute COVID-19-infection. Longitudinal follow-up of these patients is required to determine the clinical and functional outcome, treatment response and long-term effects of the SARS-CoV-2 infection.

## Introduction

The coronavirus disease 2019 (COVID-19), a manifestation of the severe acute respiratory syndrome coronavirus-2 (SARS-CoV-2), was declared a pandemic by the World Health Organization (WHO) on 11 March 2020 (1, 2). At present, the COVID-19 incidence in the United Kingdom (UK) is one of the highest in the world with 3,443,431 cases (95,675,708 globally) and 90,033 deaths (2,043,806 globally), accurate as of 19 January 2021 (3). Early reports from Wuhan, China detailed a range of neurological symptoms seen in patients with the SARS-CoV-2 infection (4). Recent isolated case reports have also described some of these manifestations, which include acute cerebrovascular disorders [CVD] (5–7), encephalopathy or encephalitis, acute demyelinating encephalomyelitis (ADEM), as well as peripheral neurological associations such as Guillain-Barre syndrome (GBS) (8, 9). Some of the proposed mechanisms underlying the increased prevalence of neurological disorders in COVID-19 include widespread systemic inflammatory and cytokine responses, diffuse intravascular coagulation and/or critical illness-related coagulopathy, direct neuronal injury, immune-mediated disorders and hemodynamic alterations (8–14).

Many reports have detailed a range of presenting neurological symptoms in patients with the SARS-CoV-2 infection, including headache, delirium, seizures, and altered mental status. However, there is often a lack of detailed description and incidence of the neurological disorders amongst hospitalized patients with COVID-19. Herein, we solely include neurological disorders, instead of non-specific neurological symptoms such as headache, dizziness and anosmia. We describe a range of neurological disorders causing neurological deficits, including their clinical, radiological and laboratory findings, encountered in our cohort of patients with COVID-19 admitted to a large tertiary institution.

## Methods

This study was registered with and approved by the East Midlands-Derby Research Ethics Committee (Ref:18/EM/0292, Major amendments) and individual patient consent was waived (15). We reviewed our prospectively collated database of all inpatient Neurology referrals, Neurology and Stroke admissions and Neurological multi-disciplinary team (MDT) meetings for all hospitalized patients with suspected or proven COVID-19 from 17 March 2020 (when national lockdown was declared in the UK) to 31 August 2020, at our institution. Each case, including the clinical, laboratory, and imaging findings, was discussed and a consensus of the underlying COVID-19 associated neurological syndrome was reached amongst the Neurology or Stroke physicians. Cases without definite neurological deficits, symptoms or signs, no clinical/radiological suspicion of COVID-19, or other more likely alternate diagnoses were excluded from our study cohort.

Patients presenting with symptoms and/or signs indicative of COVID-19 and the associated positive real-time reverse transcriptase polymerase chain reaction (RT-PCR) status from the naso-pharyngeal swab test, were classified according to the WHO COVID-19 case definition (16), into confirmed, probable and suspected cases. Further case definitions for the association of COVID-19 with neurological disease were defined based on our local MDT consensus and adapted from Ellul et al.'s compilation panel of published information (14). These included cerebrovascular disease, encephalitis, myelitis, or meningitis, and acute disseminated encephalomyelitis or acute neuropathies associated with the SARS-CoV-2 infection. Some findings of four patients from our cohort were described in a recent correspondence by the respective clinicians/authors: (Patients 21 and 22) by Hosseini et al. (17) and (Patients 18 and 28) by Dhillon et al. (18).

## Results

Our tertiary institution holds a capacity of ~1,700 hospitals beds and provides services to over 2.5 million residents. During the study period, our institution reported 1,243 COVID-19 admissions, of which 29 patients (2.3%) with neurological disorders associated with COVID-19 were identified in our study. The 29 patients included had a mean age of 68.9 ± 13.5 (SD) years, age range of 34–97 years, and there were 16 males. There were 27 Caucasian patients (92%), and only two were from the Black, Asian or Minor Ethnicity groups (BAME; one Black and one Asian). According to the WHO COVID-19 case classification, 22 patients were deemed to have confirmed COVID-19, five were probable and two had suspected COVID-19 infection. Eight patients (27%) required critical care admission, six of whom needed invasive ventilation. There was an array of neurological symptoms at presentation, namely, reduced consciousness, acute confusion, behavioral change and seizures, acute motor or sensory neurological deficits, and acute onset of movement disorders. The onset of neurological symptoms was between 9 days before to 15 days after the diagnosis or symptoms onset of COVID-19. The mean number of admission days was 25 and the range was 1–107 days. Based on the pre-defined neurological phenotype, COVID-19 patients were grouped into four main categories: 16 patients diagnosed with a cerebrovascular event (acute ischemic or hemorrhagic), seven patients with inflammatory, non-inflammatory, and autoimmune encephalopathy (including one case of transverse myelitis and two cases with known Multiple Sclerosis), three patients with movement disorders, and three patients peripheral nervous system syndromes.

### Cerebrovascular Event

#### Acute Ischaemic Stroke: (Patient 1, 2, 3, 4, 6, 10, 11, 12, 15, 16, 25, 29, 30)

**Figure 1:**
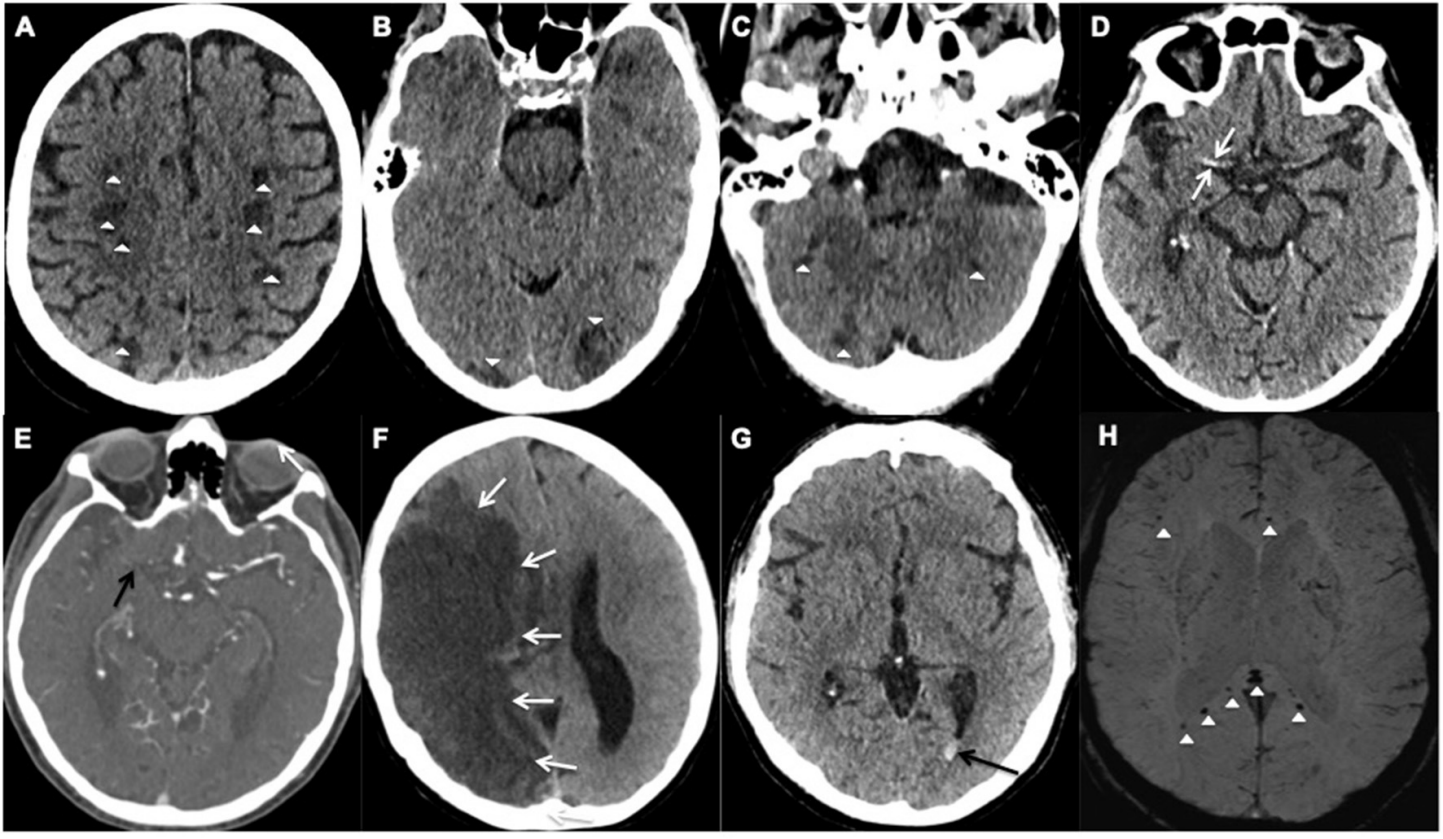
Representative examples of cerebrovascular events; **(A–F)** acute ischemic stroke and **(G,H)** hemorrhagic events. **(A–C)** Axial unenhanced CT images of Patient 28 demonstrate multifocal infarcts (white arrowheads) bilaterally along the **(A)** centrum semiovale, **(B)** Occipital lobes, and **(C)** Cerebellar hemispheres. **(D,E)** Axial CT images of Patient 25 demonstrates the **(D)** hyperdense right middle cerebral artery (MCA) sign (white arrows) and **(E)** corresponding filling defect on the CT angiogram confirming an occlusive thrombus (black arrow). **(F)** Axial unenhanced CT image of Patient 6 shows a large right MCA territory infarct (white arrows) with mass effect in keeping with an MCA malignant syndrome. **(G,H)** Axial images of Patient 18. **(G)** unenhanced CT demonstrates hyperdense layering in the occipital horn of the left lateral ventricle (black arrow) in keeping with isolated intraventricular hemorrhage (IVH), **(H)** Susceptibility weighted imaging confirms the IVH bleed within the left occipital horn (white arrow) and shows microbleeds at the splenium and genu of the corpus callosum, and subcortical white matter (white arrowheads).

Thirteen of the 28 patients (46%) with an age range of 66–97 years, and seven females, were diagnosed with acute ischemic stroke, five of which were large vessel occlusions involving the middle cerebral artery (MCA) (Table 1). Only one patient presented with a posterior circulation stroke whilst two had multifocal infarcts. All but one patient had at least one known cardiovascular risk factor. Eleven of the 13 patients had pulmonary features consistent with COVID-19. Two patients were admitted in the intensive care unit for management of a malignant MCA syndrome. All patients underwent a computed tomography (CT) and/or an magnetic resonance imaging (MRI) of the head at presentation (representative examples in Figure 1). Only two patients were given intravenous (IV) thrombolysis and two underwent mechanical thrombectomy (MT). Four patients recovered fully, three survived with disability and six patients died within days of their diagnosis due to a combination of the underlying stroke and/or COVID-19 pneumonia.

Table 1Sixteen patients with cerebrovascular events (13 Ischaemic stroke and 3 Hemorrhagic).

**Table:** 

**Patient**	**1**	**2**	**3**	**4**	**6 (Figure 1)**
Age, M/F, ethnicity, COVID-19 diagnosis	97, F, White, Definite	90,F, White, Definite	66,F, White, Definite	85, M, White, Probable	71, M, White, Definite
Stroke type, Thrombolysis/ Thrombectomy	Left ICA/MCA infarct; Thrombolysis	Left partial anterior circulation stroke (PACS)	Left ICA/MCA infarct; Mechanical thrombectomy	Right MCA infarct; Thrombolysis	Right MCA infarct
Presenting symptoms/signs	Right hemiparesis	Right hemiparesis and dysphasia	Right hemiparesis, hemianopia and dysphasia	Left hemiparesis	GCS 3
Blood results at admission:	Hemoglobin 136 g/l lymphocyte count 1.39 neutrophil count 3.41 platelet count 207 CRP 6 mg/L D-dimer; NR Ferritin; NR Creatinine 66 umol/L PT 12.6 s	Hemoglobin 125 g/l lymphocyte count 0.98 neutrophil count 10.98 platelet count 421 **CRP 276** mg/L **ESR mm/h: 116** D-dimer; NR Ferritin; NR Creatinine 59 umol/L PT 11.1 s	Hemoglobin 131 g/l lymphocyte count 2.91 neutrophil count 9.74 platelet count 400 **CRP 62** mg/L **D-dimer ug/l; 3144** Ferritin; NR Creatinine 64 umol/L PT 11.6 s	Hemoglobin 140 g/l **lymphocyte count 0.92** neutrophil count 5.53 platelet count 231 **CRP 23** mg/L D-dimer; NR Ferritin; NR Creatinine 89 umol/L PT 11.9 s	Hemoglobin 106 g/l **lymphocyte count 0.15** neutrophil count 2.81 **platelet count 38** **CRP 298** mg/L D-dimer; NR Ferritin; NR Creatinine 119 umol/L PT 11.9 s
Brain Imaging	CT: Acute thrombus within the left ICA and MCA consistent with an acute infarct	CT: No acute abnormality. Severe small vessel disease	CT: Left MCA territory infarct, with associated mass effect	CT: Right MCA territory infarct	CT: Massive acute right MCA territory infarct with mass effect and shift of midline structures
Outcome status	Died	Full recovery	Continued recovery in rehabilitation; mRS 5	Full recovery	Died
**10**	**11**	**12**	**15**	**16**	**25**
88, F, White, Definite	69, F, White, Probable	78, M, White, Suspected	66, M, White, Definite	89, M, White, Definite	78, F, White, Definite
Right PCA Infarct	Left ACA infarct	Right MCA infarct	Right MCA infarct	Right MCA/ACA infarct	Left MCA infarct; Mechanical Thrombectomy
Left hemiparesis	Right hemiparesis and dysphasia	Left hemiparesis and confusion	GCS 3	Left hemiparesis	Right hemiparesis and dysphasia
Hemoglobin 113 g/l **lymphocyte count 0.80** neutrophil count 6.82 platelet count 296 **CRP 30** mg/L D-dimer; NR Ferritin 126 ug/l Creatinine 62 umol/L PT; NR	Hemoglobin 108 g/l **lymphocyte count 0.76** neutrophil count 11.27 platelet count 196 **CRP 74** mg/L D-dimer; NR Ferritin; NR Creatinine 34 umol/L PT 12.1 s	Hemoglobin 145 g/l **lymphocyte count 0.49** neutrophil count 8.14 platelet count 332 **CRP 132** mg/L D-dimer; NR Ferritin 38 ug/l Creatinine 81 umol/L PT 13 s	Hemoglobin 117 g/l **lymphocyte count 0.87** neutrophil count 10.72 platelet count 352 **CRP 296** mg/L D-dimer; NR Ferritin; NR Creatinine 84 umol/L PT 10.9 s	Hemoglobin 136 g/l **lymphocyte count 0.82** neutrophil count 7.87 platelet count 335 **CRP 345** mg/L D-dimer; NR Ferritin; NR **Creatinine 118** umol/L PT 14.4 s	Hemoglobin 105 g/l lymphocyte count 1.19 neutrophil count 8.49 platelet count 362 CRP 8 mg/L D-dimer; NR Ferritin; NR **Creatinine 106** umol/L PT 12.6 s
MRI: Acute right PCA infarct	CT: Acute left ACA territory infarct	CT: Small cortical infarcts involving the right pre-central gyrus	CT: Large acute right MCA territory infarct with mass effect	CT; Acute thrombus within the terminal segment ICA, M1/M2 segments of the right MCA and the proximal right A1 ACA segment	CT: Left MCA thrombus and infarct
Continued recovery in rehabilitation; mRS 3	Full recovery	Continued recovery in rehabilitation; mRS 3	Died	Died	Full recovery

**Table:** 

**28 (****Figure 1****)**	**18 (****Figure 1****)**	**24**	**29**	**30**
65, M, White, Definite	49, M, White, Definite	56, F, Black, Suspected	34, F, White, Definite	68, M, Definite
Isolated intraventricular hemorrhage	1. Isolated Intraventricular hemorrhage 2. Unilateral hearing loss (new)	Cerebral microbleeds, suspected CAA	Multifocal cerebral and cerebellar infarcts	Multifocal cerebral and cerebellar infarcts
Reduced consciousness despite sedation hold	Reduced consciousness despite sedation hold	Recurrent seizures	Dysphasia and dysarthria	GCS 3
Hemoglobin 127 g/l **lymphocyte count 0.17** neutrophil count 3.99 platelet count 228 **CRP 158** mg/L **D-dimer 8645** ug/l **Ferritin 758** ug/l **Creatinine 128** umol/L PT 10.8 s	Hemoglobin 141 g/l **lymphocyte count 0.52** neutrophil count 3.51 platelet count 170 **CRP 171** mg/L D-dimer; NR **Ferritin 2132** ug/l Creatinine 91 umol/L PT 11.4 s	Hemoglobin 147 g/l **lymphocyte count 0.86** neutrophil count 6.02 platelet count 202 CRP <5 mg/L D-dimer; NR Ferritin; NR Creatinine 95 umol/L PT 12.9 s	Hemoglobin 91 g/l **lymphocyte count 0.94** neutrophil count 5.87 platelet count 455 **CRP 263** mg/L **D-dimer 6358** ug/l **Ferritin 2986** ug/l **Creatinine 286** umol/L PT 11.4 s	Hemoglobin 132 g/l **lymphocyte count 0.66** neutrophil count 9.35 platelet count 412 **CRP 342** mg/L D-dimer; NR **Ferritin 3712** ug/l Creatinine 75 umol/L **PT 13.1** s
CT; Small volume intraventricular hemorrhage in the left occipital horn of the lateral ventricle	CT and MRI: Small volume intraventricular hemorrhage layering in both occipital horns	MRI; Micro-hemorrhagic changes in both cerebral hemispheres	CT and MRI: Multiple bilateral white matter and left cerebellar tiny foci of infarction	CT; Multifocal supra- and infra-tentorial infarcts
Full recovery	Recovery with slight disability; mRS 1	Full recovery	Died	Died

*M, male; F, female; ICA, internal carotid artery; ACA, anterior cerebral artery; MCA, middle cerebral artery; PCA, posterior cerebral artery; GCS, Glasgow Coma Scale; CAA, cerebral amyloid angiopathy; NR, no result; CRP, C-Reactive protein; ESR, Erythrocyte sedimentation rate; mRS, modified Rankin Score; PT, Prothrombin time; CT, computed tomography. MRI, magnetic resonance imaging; Lymphocyte, neutrophil and platelet count; numbers x 10 E9/L. The bold values highlight abnormal values*.

#### Hemorrhagic Stroke: (Patient 18, 24, 28)

Two male patients with COVID-19, Patients 18 and 28 (aged 48 and 65 years respectively), had isolated intraventricular hemorrhage demonstrated on the CT scan of the head, performed due to reduced level of consciousness despite a sedation hold, during their prolonged critical care admission (Table 1). The MRI of the head from the 48-year-old patient showed cerebral microbleeds in the splenium of the corpus callosum and subcortical white matter (Figure 1). Both patients were placed on continuous veno-venous hemofiltration, but neither required extracorporeal membrane oxygenation (ECMO). The platelet count level, prothrombin and activated partial thromboplastin times were within the normal referenced ranges.

The third patient (Patient 24) was a 55 year-old female who presented with recurrent seizures. The MRI of the head also revealed cerebral microbleeds, with features suggestive of cerebral amyloid angiopathy. No critical care admission was required and the prothrombin time was normal. Two patients (Patient 24 and 28) made an uneventful recovery. However, patient 18 reported unilateral hearing impairment following hospital discharge.

### Inflammatory, Non-inflammatory, and Autoimmune Encephalopathy

Limbic encephalitis (Patient 21); Inflammatory encephalopathy (Patient 22); ADEM (Patient 27); Transverse myelitis (Patient 5); Non-inflammatory encephalopathy (Patient 9) (Table 2).

**Table 2:** Seven patients with inflammatory, non-inflammatory or autoimmune encephalopathy, including two patients with known multiple sclerosis (MS), and one patient with transverse myelitis.

**Patient**	**27 (Figure 2)**	**21 (Figure 2)**	**22**	**5**	**9**	**8**	**14**
Age, M/F, ethnicity, COVID-19 diagnosis	68, F, White, Definite	79, F, White, Definite	46, M, Asian, Definite	69, M, White, Probable	55, F, White, Definite	65, M, White, Definite	56, M, White, Definite
Final neurological diagnosis (impression)	Acute demyelinating encephalomyelitis (ADEM)	Limbic encephalitis associated with SARS-CoV2	Inflammatory encephalopathy associated with SARS-CoV2	Transverse myelitis	Non-inflammatory encephalopathy associated with SARS-CoV2	Relapse in an advanced Secondary Progressive MS	Relapse in a known Multiple Sclerosis
Key neurological signs	Delirium, limb weakness, ataxia, visual hallucination,	Delirium, New onset generalized seizure/status epilepticus, dysphagia, cognitive impairment, amnesia	Delirium, New onset generalized seizures, disinhibition and cognitive impairment	Quadriparesis and sensory loss at cervical level	Headache, delirium, reduced conscious level, confusion and behavioral change	Delirium, reduced consciousness	Worsening limb weakness and dysarthria
Blood results at admission	Hemoglobin 124 g/l **Lymphocyte count 0.55** Neutrophil count 9.11 Platelet count 336 **CRP 262 mg/L** ESR 10 mm/hr D-dimer; NR Ferritin; NR Creatinine 94 umol/L PT 10 s	Hemoglobin 132 g/l **Lymphocyte count 0.39** Neutrophil count 5.83 Platelet count 313 **CRP 31** mg/L **D-dimer 3748** ug/l Ferritin; NR Creatinine 65 umol/L PT 13.7 s	Hemoglobin 147 g/l **Lymphocyte count 0.89** Neutrophil count 3.6 Platelet count 133 **CRP 149** mg/L **ESR 90** mm/hr **D-dimer 1659** ug/l **Ferritin; 1328** ug/l Creatinine 88 umol/L PT 11.9 s	Hemoglobin 120 g/l Lymphocyte count 1.1 Neutrophil count 8.9 Platelet count 407 **CRP 24 mg/L** D-dimer; NR Ferritin; NR Creatinine 53 umol/L PT; NT	Hemoglobin 132 g/l Lymphocyte count 1 Neutrophil count 9.1 Platelet count 203 **CRP 47 mg/L** D-dimer; NR Ferritin; NR Creatinine 67 umol/L PT; NR	Hemoglobin 147 g/l **Lymphocyte count 0.46** Neutrophil count 6.4 Platelet count 154 **CRP 168** mg/L **D-dimer 3748** ug/l Ferritin; NR Creatinine 69 umol/L PT 10.4 s	Hemoglobin 132 g/l **Lymphocyte count 0.76** Neutrophil count 3.67 Platelet count 153 **CRP 62** mg/L **D-dimer 5422** ug/l Ferritin 381 ug/l Creatinine 64 umol/L PT 11.2 s
Brain imaging	MRI; Multiple patchy, asymmetric periventricular and subcortical white matter lesions in bilateral cerebral hemispheres, midbrain, dorsal pons, right middle cerebellar peduncle, medulla and right cerebellar hemisphere. Mixed diffusivity exhibited. Radiological progression on subsequent imaging during hospital admission	MRI; Lesions in the limbic system, predominantly in the left amygdala and hippocampus with partial restricted diffusion	MRI; White matter lesions in the left anterior limbic structures with foci of increased diffusivity suggesting cellular inflammation	MRI; Restricted diffusion in the inferior medulla with enhancement. Small area of restricted diffusion in left middle cerebellar peduncle. MRI spine; Extensive spinal cord abnormality predominantly involving the cervical and lower thoracic regions including the conus in keeping with transverse myelitis	MRI; No acute abnormality. Marked parenchymal atrophy with medial bitemporal predominance	CT: No acute abnormality	MRI: Progression of inflammatory demyelinating plaques since 2015
CSF examination	White cells 3 per μl (first day of admission), White cells 8 lymphocytes per μl (14 days after admission), red cells 1, **protein 45.1 mg/dl**, glucose 4.1, Paired serum glucose 5.6 mmol/L. **Oligoclonal bands** present. Viral PCR for SARS-CoV-2/enterovirus/HSV negative. NMDA-R/CASPR2/LGi-1/Glycine receptor antibodies all negative, paraneoplastic anti-neuronal antibodies negative, anti-MOG and AQP4 negative	White cell none, Red cells 9, protein; protein 340 mg/dl, glucose 4.1, Paired serum glucose 5.4 mmol/L. Oligoclonal bands negative. Viral PCR for SARS-CoV-2/enterovirus /HSV negative. NMDA-R/CASPR2/LGi-1/Glycine receptor antibodies all negative, paraneoplastic anti-neuronal antibodies negative	White cell none, Red cells 25, **protein 98.7 mg/dl**, glucose 4.5, Paired serum glucose 5.8 mmol/L. **Oligoclonal bands** present. Viral PCR for SARS-CoV-2/enterovirus /HSV negative. NMDA-R/CASPR2/LGi-1/Glycine receptor antibodies all negative, paraneoplastic anti-neuronal antibodies negative	White cell 1, red cells 0, **protein 51.4 mg/dl**, glucose 3.1 mmol/L. **Oligoclonal bands** present (identical to serum). Viral PCR for SARS-CoV-2/enterovirus /HSV negative. NMDA-R/CASPR2/LGi-1/Glycine receptor antibodies all negative, paraneoplastic anti-neuronal antibodies negative	Not performed (known early-onset advanced Alzheimer's Dementia with further cognitive decline)	Not performed	Not performed
Neurological treatments; recovery	Corticosteroids; Intravenous Methylprednisolone	Benzodiazepine, Levetiracetam IV & maintenance	Intubation and sedation for status epilepticus, Empirical aciclovir, antibiotics until CSF results available. IV valproate & maintenance	Intravenous Methylprednisolone	Supportive	Supportive and antibiotics (due to advanced disorder, osteomyelitis and pressure sores)	Glatiramer acetate injections 3 times/weekly
Outcome status	Recovery with mild neurological deficits; mRS 1	Partial recovery with mild neurological deficits; mRS 1	Full clinical recovery	Partial recovery with neurological deficits; Moderate disability mRS 4	Partial recovery with neurological deficits; Severe disability mRS 5	Died	Partial recovery with moderate disability; mRS 3

*M, male; F, female; NR, no result; CRP, C-Reactive protein; mRS, modified Rankin Score; ESR, Erythrocyte sedimentation rate; PCR, polymerase chain reaction; NMDA-R, N-methyl-D-aspartate Receptor antibodies; LGi-1, Leucine-rich glioma inactivated-1; CASPR2, contactin-associate protein-like 2; MOG, myelin oligodendrocyte glycoprotein; AQP4, Aquaporin-4; PT, Prothrombin time. Paraneoplastic anti-neuronal antibodies including GABA-B; γ-Aminobutyric acid-B receptor; AMPA; GluR1 and GluR2 subunits of the AMPA receptor. CT, computed tomography. MRI, magnetic resonance imaging, Lymphocyte, neutrophil and platelet count, numbers x 10 E9/L. The bold values highlight abnormal values*.

Three patients (aged between 46 to 79 years, three females) demonstrated a range of inflammatory encephalopathies. Patient 21 was diagnosed with limbic encephalitis, patient 22 with inflammatory encephalopathy, patient 27 with ADEM. Patient 9 was diagnosed with non-inflammatory encephalopathy and Patient 5 with transverse myelitis. Whilst four patients (9, 21, 22, and 27) presented with acute-onset delirium and altered mental status, patients 21 and 22 also suffered from status epilepticus, cognitive impairment (scored 19 and 20 on the Montreal Cognitive Assessment, respectively) and amnesia. Patient 5 presented with quadriparesis and altered sensation at the cervical level. In Patient 27, three white cells were found in the cerebrospinal fluid (CSF) on the first day of admission, prior to commencement of steroid treatment. A repeat lumbar puncture on Day 14 was performed after a course of corticosteroid treatment (intravenous Methylprednisolone 1 gram per day for three days followed by oral Prednisolone 60 milligrams per day). The second CSF examination revealed 8 lymphocytes per μl. There was no evidence of pleocytosis in Patient 21 (12 days after admission) or Patient 22 (two days after admission). Oligoclonal bands and mildly raised proteins were detected in the CSF of Patients 5, 22, and 27, whilst intrathecal SARS-CoV-2, paraneoplastic and autoimmune encephalitis antibodies (including N-methyl-D-aspartate Receptor, Leucine-rich glioma inactivated-1, contactin-associate protein-like 2, γ-Aminobutyric acid-B receptor, GluR1 and GluR2 subunits of the AMPA receptor) were negative in all three patients. Patient 9 had a pre-morbid diagnosis of Early Onset Alzheimer's Dementia, and missed out on a diagnostic lumbar puncture due to the time's real-life challenges and the guidelines that were based on uncertainties and lack of knowledge on SARS-CoV2 transmission routes.

**Figure 2:**
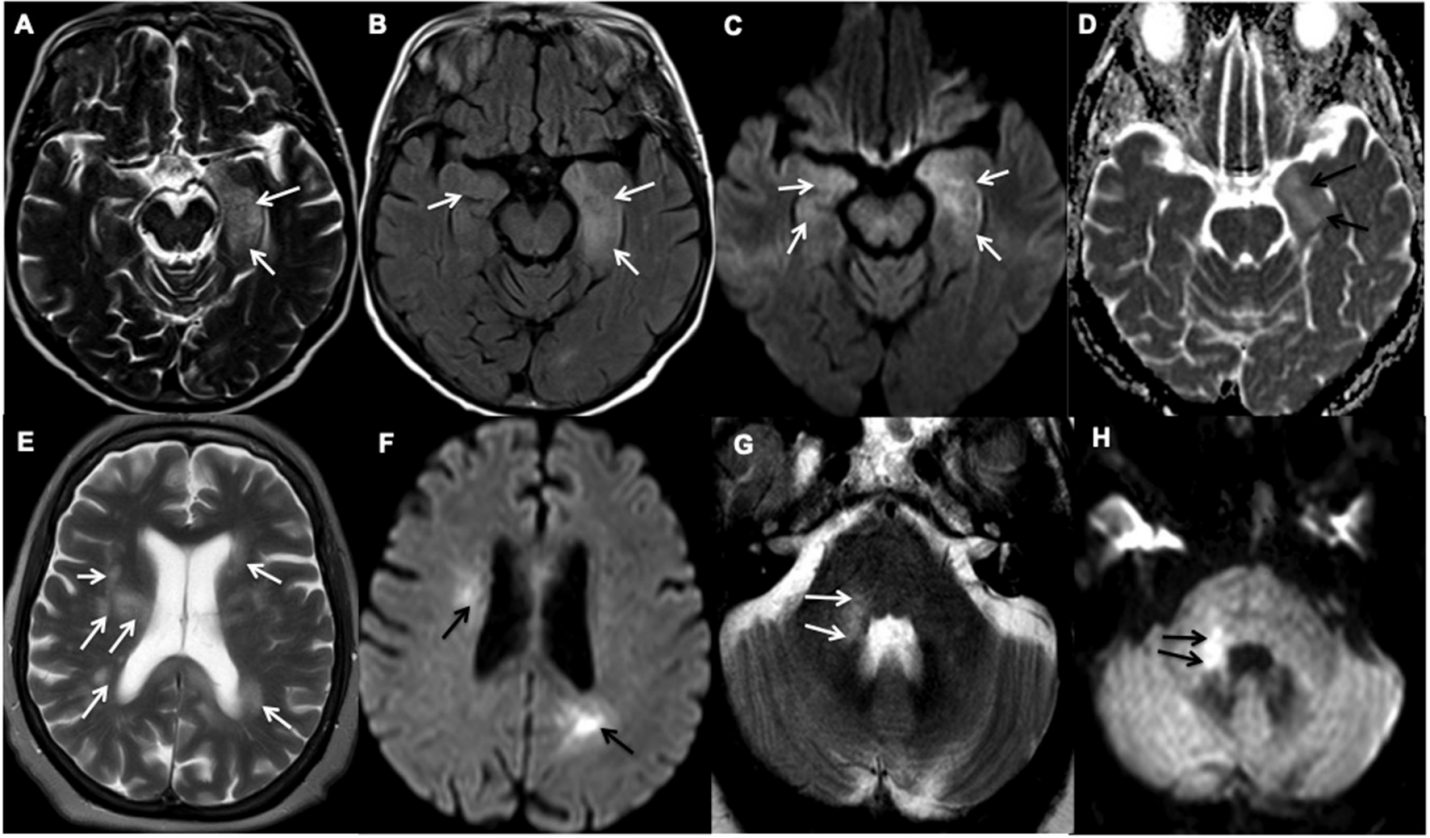
Representative examples of inflammatory encephalopathy. **(A–D)** Axial MR images of Patient 21 demonstrate limbic encephalitis. **(A)** T2-weighted and **(B)** Fluid attenuated inversion recovery (FLAIR) imaging demonstrate hyperintensity in both medial temporal lobes, but predominantly in the left amygdala and hippocampus (white arrows), **(C)** Diffusion-weighted imaging (white arrows) and **(D)** Apparent diffusion coefficient (ADC) show corresponding partial diffusion restriction (black arrows). **(E–H)** Axial MR images of Patient 27 demonstrate acute demyelinating encephalomyelitis. **(E)** T2-weighted imaging demonstrates patchy and asymmetric white matter hyperintensities within the periventricular and subcortical regions and **(G)** right middle cerebellar peduncle (white arrows). **(F,H)** Diffusion-weighted imaging shows corresponding high diffusion signal (black arrows).

The MRI of the head showed partial diffusion restriction in the limbic system of Patient 21 (Figure 2) and persistent diffusion-weighted hyperintensities without overt restriction in Patient 22, suggestive of cellular inflammation. Patient 27 had progressive patchy, asymmetric periventricular and subcortical white matter hyperintense foci with diffusion restriction in the cerebral hemispheres, right cerebellar hemisphere and brainstem, which are features consistent with ADEM (Figure 2). The MRI of the spine in Patient 5 showed extensive spinal cord abnormality involving the cervical, lower thoracic, and conal regions that were supportive of transverse myelitis. Small regions of diffusion restriction were also identified in the medulla and middle cerebellar peduncle. Patient 22 required a brief critical care admission for four days to control seizures, Patients 5 and 27 were given a course of intravenous (IV) followed by oral corticosteroids. Patient 22 was considered, but did not receive corticosteroid treatments as he had a rapid recovery after seizure-control and was discharged from the intensive care unit. During the first peak of COVID-19 in the UK, the evidence on the use of corticosteroids during acute phase of SARS-CoV-2 infection, particularly during intensive care admissions, was controversial (19). All five patients recovered from their acute respiratory illness sufficiently for hospital discharge. However, only Patient 22 made a full neurological recovery, whilst the remaining patients were identified as having persistent neurological disability at discharge.

#### Demyelination: (Patient 8, 14)

Two male patients (Patients 14 and 8), with known advanced Multiple Sclerosis (MS) (aged between 56 to 65 years, respectively), presented with symptoms consistent with, and were diagnosed with COVID-19 (Table 2). Patient 8 presented with acute delirium whilst Patient 14 was hospitalized due to the MS relapse associated with worsening of his baseline MS-related limb weakness and dysarthria during the admission. The MRI of the head performed showed progression of the inflammatory plaques since 2015 in Patient 14 but no acute changes were identified on the CT scan of the head obtained from Patient 8. None of them were admitted to the intensive care unit. Only Patient 14 survived with ongoing disability, whilst Patient 8 succumbed to septicaemia.

### Movement Disorders

#### Myoclonus ± Opsoclonus (Patients 7, 19, 20)

Movement Disorders were seen in three patients with COVID-19 (Patients 7, 19 and 20; aged between 57 to 86 years, two males) (Table 3). Patient 7 suffered from mild rest myoclonus of the arm, Patient 20 had a generalized rest myoclonus and Patient 19 was diagnosed with opsoclonus-myoclonus syndrome. No other neurological symptoms or signs were identified. The CT/MRI of the head demonstrated no acute abnormality in all patients. There was elevated CSF protein in Patients 19 and 20, with otherwise normal CSF constituents (including no detectable intrathecal SARS-CoV-2). Normal electroencephalography (EEG) record was demonstrated in Patient 20. All patients were treated with benzodiazepines and two patients made a full recovery at the time of discharge from hospital while Patient 19 had residual symptoms. Only Patient 7 required critical care admission for pulmonary symptoms of COVID-19.

**Table 3:** Three patients with movement disorders.

**Patient**	**7**	**19**	**20**
Age, M/F, ethnicity, COVID-19 diagnosis	57, M, White, Definite	86, F, White, Definite	86, M, White, Probable
Final neurological diagnosis	Myoclonus	Opsoclonus myoclonus	Generalized rest myoclonus affecting lower face and whole body
Blood results at admission	Hemoglobin 104 g/l **Lymphocyte count 0.56** Neutrophil count 3.22 Platelet count 223 **CRP 409** mg/L **D-dimer; 17390** **Ferritin; 916** Creatinine 84 umol/L PT 12.5 s	Hemoglobin 109 g/l **Lymphocyte count 0.86** Neutrophil count 10.2 Platelet count 271 CRP 5 mg/L D-dimer; NR Ferritin; NR Creatinine 80 umol/L PT; NR	Hemoglobin 97 g/l **Lymphocyte count 0.76** Neutrophil count 4.51 Platelet count 302 **CRP 35** mg/L**, ESR 105** mm/hr D-dimer; NR Ferritin; NR Creatinine 139 umol/L PT; NR
Brain imaging	CT: No acute abnormality	MRI: No acute abnormality	MRI: No acute abnormality Electroencephalography: Normal
CSF examination	Not performed	White cell none, red cell none, protein; 46.8 mg/dl, glucose; 3.9 mmol/L. paired serum glucose; NR. Viral PCR for SARS-CoV-2/enterovirus/HSV negative. NMDA-R/CASPR2/LGi-1/Glycine receptor antibodies all negative	White cell none, red cells 265, **protein; 166.7 mg/dl**, glucose 3.6 mmol/L, paired serum glucose; NR. **Oligoclonal bands** present in serum and CSF. Viral PCR for SARS-CoV-2/enterovirus/HSV negative. NMDA-R/CASPR2/LGi-1/Glycine receptor antibodies all negative
Neurological treatments; recovery	Diazepam	Levetiracetam and clonazepam	Clonazepam
Outcome status	Full recovery	Partial recovery with neurological deficits; Moderate disability mRS 3	Full recovery

*M, male; F, female; NR, no result; CRP, C-Reactive protein; mRS, modified Rankin Score; NMDA-R, N-methyl-D-aspartate Receptor antibodies; ESR, Erythrocyte sedimentation rate; PCR, polymerase chain reaction; LGi-1, Leucine-rich glioma inactivated-1; CASPR2, contactin-associated protein-like 2. PT, Prothrombin time; CT, computed tomography. MRI, magnetic resonance imaging, Lymphocyte, neutrophil and platelet count, numbers x 10 E9/L. The bold values highlight abnormal values*.

### Peripheral Nervous System Disorders

#### Acute Inflammatory Demyelinating Polyneuropathy (Patient 13, 23); Brachial Plexopathy (Patient 26)

Two patients with COVID-19 (Patient 13 and 23) presented with ascending peripheral weakness and diagnosed with acute inflammatory demyelinating polyneuropathy or Guillain-Barré syndrome (Table 4). The ascending distal limb weakness in Patient 23 was associated with seizures and an atypical acute inflammatory demyelinating polyneuropathy was diagnosed. The nerve conduction studies confirmed features in keeping with segmental demyelinating peripheral neuropathy in Patient 13 and axonal neuropathy (motor and sensory) in Patient 23. Elevated CSF protein with otherwise normal CSF constituents was identified in both these patients. Patient 26 who presented with left upper limb weakness was diagnosed with brachial plexopathy.

**Table 4:** Three patients with disorders of the peripheral nervous system.

**Patient**	**13**	**23**	**26**
Age, M/F, ethnicity, COVID-19 diagnosis	67, M, White, Definite	58, F, White, Probable	58, M, White, Definite
Final neurological diagnosis	Guillain-Barré syndrome	Atypical acute inflammatory demyelinating polyneuropathy	Brachial plexopathy 2. Bilateral hearing loss
Blood results at admission	Hemoglobin 133 g/l **Lymphocyte count 0.93** Neutrophil count 3.81 Platelet count 242 **CRP 112** mg/L D-dimer; NR Ferritin; 295 Creatinine 58 umol/L PT; NR	Hemoglobin 85 g/l **Lymphocyte count 0.48** Neutrophil count 5.19 Platelet count 178 **CRP 16** mg/L D-dimer; NR Ferritin 340 ug/l **Creatinine 140** umol/L PT 11.4 s	Hemoglobin 101 g/l Lymphocyte count 1.24 Neutrophil count 16.36 Platelet count 224 **CRP 351** mg/L **D-dimer 9251** ug/l **Ferritin 800** ug/l Creatinine 62 umol/L PT 11.5 s
Brain imaging	Not performed	MRI Brain and spinal cord: No acute abnormality	CT brain: No acute abnormality
Nerve conduction study	Segmental demyelinating peripheral neuropathy	Sensory and motor axonal neuropathy	Not performed during the acute SARS-CoV2 infection
CSF examination	Red cells: 1770, white cells: none; **protein; 155.6 mg/dl**, glucose; 4.2, Paired blood glucose; 6.6 mmol/L; Viral PCR for enterovirus/HSV negative	Red cells 8; White cells: none, **protein; 340.1 mg/dl**, glucose; 4.2, Paired blood glucose; 6.6 mmol/L; Oligoclonal bands negative. Viral PCR for SARS-CoV-2/enterovirus/HSV negative. NMDA-R/CASPR2/LGi-1/Glycine receptor antibodies all negative	Not performed
Neurological treatments; recovery	Intravenous Immunoglobulin	Intravenous Methylprednisolone, Levetiracetam & Sodium Valproate (seizures)	No specific treatment administered (neurological disorder identified after ventilation support in critical care)
Outcome status	Recovery with slight disability; mRS 1	Partial recovery with neurological deficits; mRS 4	Partial recovery with slight disability; mRS 2

*M, male; F, female; NR, no result; CRP, C-Reactive protein; mRS, modified Rankin Score; NMDA-R, N-methyl-D-aspartate Receptor antibodies; PCR, polymerase chain reaction; LGi-1, Leucine-rich glioma inactivated-1; CASPR2, contactin-associated protein-like 2. PT, Prothrombin Time; CT, computed tomography. MRI, magnetic resonance imaging; Lymphocyte, neutrophil and platelet count, numbers x 10 E9/L. The bold values highlight abnormal value*.

Patient 13 was treated with intravenous immunoglobulin (IVIG). Patients 23 and 26 required a short period of critical care admission and all patients made a partial recovery. Patient 26 reported bilateral hearing loss following hospital discharge and has been referred for further investigation.

## Discussion

We report a variety of neurological disorders with a clinical impact in patients with COVID-19 infection admitted in a large tertiary institution during the “first wave” of the COVID-19 pandemic in the UK. There have been isolated reports of various neurological disorders associated with previous outbreaks of the severe acute respiratory syndrome (SARS) and Middle East acute respiratory syndrome (MERS) (20, 21). Similarly, our cohort of cases demonstrates a wide range of COVID-19 related neurological disorders, ischemic and hemorrhagic cerebrovascular events, inflammatory and non-inflammatory encephalopathy syndromes, transverse myelitis, movement disorders, and acute inflammatory demyelinating polyneuropathy with additional neurological manifestations. Excluding anosmia, the cumulative incidence of disorders is in 2.3% of our hospitalized patients with COVID-19, with 27% of them requiring critical care admission. The diagnosis of each of the neurological disorders was made in conjunction with a positive diagnosis of COVID-19, suggesting their association may not be fortuitous. Whilst recent case reports/series have described a range of neurological symptoms during the ongoing COVID-19 pandemic, there is often a lack of detailed clinical, radiological and laboratory findings of the neurological disorders amongst hospitalized patients with COVID-19, which reflect the challenge of studying the natural history of COVID-19 complications in this patient cohort.

Evidence from our cohort and recent studies have included non-specific initial presentations such as altered mental status or delirium, features commonly seen in the critically unwell with sepsis and hypoxemia, as well as being potential early signs of dementia. The neurological disorders have been reported in patients who present solely with neurological signs and symptoms as well as those with established systemic or pulmonary illness related to COVID-19. These neurological features may precede or occur days after the onset of pulmonary symptoms. Hence, the variable and non-specific nature of the presentation and onset of the illness creates a diagnostic and therapeutic dilemma. Furthermore, the occasional delay of presentation and hospital admission during the first peak of the pandemic due to patients' fear, isolation or shielding, may have lead to an increase in severity of the illness and neurological disorders at the point of diagnosis. Interestingly, there was no increased incidence or severity of the COVID-19 infection or neurological disorders amongst the BAME groups in our cohort. However, this could be due to the relatively small number of BAME communities in our geographical region. Additionally, during the first wave that peaked in May 2020, patients with acute SARS-CoV-2 infection did not receive corticosteroids or anti-viral therapy as part of standard treatment. Whilst some patients may have been recruited to the RECOVERY trial (22), which included dexamethasone and anti-viral therapy arms, none of the patients in this case series were involved.

There has been a reported increase in the incidence and severity of cerebrovascular disease associated with COVID-19, particularly in a younger cohort (6, 23). Our cohort demonstrated a high percentage of patients with acute ischemic stroke and up to 38% of these had a large vessel occlusion. Some of the acute ischemic cerebrovascular events with multifocal infarcts may be cardioembolic in nature, due to associated cardiovascular risk factors, but coagulopathy, vasculitis and viral endothelialitis have also been reported as potential causes of multi-vessel stroke in patients with COVID-19 (23, 24). The hyper-inflammatory syndrome or “cytokine storm” strongly associated with severe COVID-19 infection could also contribute to the underlying etiology (13).

Thrombotic microangiopathy and endothelial dysfunction, also evident in multiple organ systems related to COVID-19, may be contributory factors in sepsis/critical illness-related cerebral microbleeds (24, 25). The SARS-CoV-2 has been shown to preferentially bind to the angiotensin converting enzyme (ACE)-2 receptors that can be found in the endothelial lining, leading to the breakdown of the blood-brain barrier (8). However, cerebral microbleeds have similarly been reported in acute respiratory distress syndrome patients with a resemblance seen in cerebral microbleeds-related high altitude exposure, sharing a common underlying etiology of hypoxemia (26). This could likewise explain the findings in our cases with hemorrhagic neurological manifestations in COVID-19. Interestingly, both patients with isolated intraventricular hemorrhage had normal coagulation parameters, and the observed cerebral microbleeds were atypical for hypertensive or amyloid angiopathy causes. Other variables that may influence the presence and/or extent of microhemorrhage in patients with COVID-19 include therapeutic anticoagulation and raised cerebral venous pressure secondary to ventilator measures in optimizing patient oxygenation in the critical care setting (18).

The neurotropic potential of COVID-19 via direct viral axonal injury has been alluded to following scarce reports of the SARS-CoV-2 being detected in the CSF of patients with meningo-encephalitis and in animal models (27, 28). Similarly, a large case series of brain autopsies in patients with COVID-19 revealed the detection of the SARS-CoV-2 RNA and proteins in up to 53% of patients, although its presence was not associated with the severity of the immuno-pathological findings (29). Furthermore, few case reports have demonstrated imaging features of direct neuronal injury of the olfactory pathway in COVID-19 patients presenting with anosmia, adding strength to this potential mechanism (30). Nonetheless, no detectable intrathecal coronavirus strain was identified in our patients who presented with encephalopathy. Recent CSF-based studies in patients with COVID-19 also failed to detect any evidence of SARS-CoV-2 intrathecally (31). However, it remains unclear if this reflects the poor sensitivity of the RT-PCR assay in CSF resulting in a possible false negative result, or if other (indirect) immune-mediated mechanisms are responsible for the neurological changes (32).

Interestingly, two patients who required critical care admission reported new onset hearing loss following hospital discharge, despite no acute abnormality on their MRI of the head during the hospital stay. A recent case report also described a possible association between sensorineural hearing impairment and COVID-19 in the critical care setting (33). It is postulated that such an observation could be due to the underlying hyperinflammatory process and/or the neuro-invasive potential of the SARS-CoV-2 against the auditory nervous system. Hence, it will be important to consider screening patients with severe COVID-19 infection for hearing impairment during the hospital admission.

An immunological response secondary to the SARS-CoV-2, resulting in cerebral inflammation and edema with clinical encephalopathic features may offer an alternative explanation for the incidence of inflammatory and auto-immune encephalopathy disorders (14, 34). Antibodies against neuronal synaptic proteins have been demonstrated in autoimmune encephalitis, and there have been increased numbers of antibodies reported against other coronavirus strains, suggesting a possible association between auto-immune or inflammatory encephalopathic disorders and the COVID-19 infection (35, 36). Furthermore, the presence of both intrathecal and serum oligoclonal bands in two patients with acute encephalopathy and a patient with ADEM suggests that the immune-mediated response is not restricted to the intrathecal production of immunoglobulins. Post-infectious autoimmune disorder is also demonstrated in our case cohort of acute inflammatory demyelinating polyneuropathy, whereby the onset of neurological symptoms followed an initial period of illness related to COVID-19. Expected electrophysiological changes in keeping with demyelinating peripheral neuropathy were confirmed in one patient and the anticipated response to the IV immunoglobulin therapy was observed.

Limitations of our study include its lack of pathological evidence to prove causality. Furthermore, we only included hospitalized patients with COVID-19 in our study, thereby potentially underestimating the true incidence of the neurological associations in patients in the community. There were also inherent drawbacks in the sensitivity and specificity of the available RT-PCR swab tests during the study period, which may have underestimated the incidence of COVID-19 in the patient population (37).

Although the exact mechanism and possible causality of the SARS-CoV-2 infection associated with each of the presented neurological disorders remains unclear, it is likely that shared pathophysiological mechanisms are responsible for the various neurological manifestations of COVID-19. Our study lends further support to the growing body of evidence, aiding better understanding of the neurological features and optimizing management strategies using an approach guided by the evolution of clinical, laboratory and imaging features. Longitudinal follow-up of these patients is required to determine the long term effects, treatment response and outcome of the SARS-CoV-2 infection.

## Data Availability Statement

The original contributions presented in the study are included in the article/supplementary materials, further inquiries can be directed to the corresponding author/s.

## Ethics Statement

The studies involving human participants were reviewed and approved by East Midlands-Derby Research Ethics Committee (Ref:18/EM/0292, Major amendment). The Ethics Committee waived the requirement of written informed consent for participation.

## Author Contributions

AH conceptuated and designed the study, prospectively screened, identified, collected and registered patients with neurological COVID-19. AH and RD held multidisciplinary meetings to discuss patients with COVID-19. RT and EN registered a prospective clinical audit to identify patients. PD and AH applied for ethical approval. PD, AH, HM, and RT obtained clinical information from medical records. AH, RT, and CC described neurological phenotype. PD described radiological features and wrote the manuscript. AH edited versions and revised the final manuscript. All co-authors edited the manuscript.

## Conflict of Interest

The authors declare that the research was conducted in the absence of any commercial or financial relationships that could be construed as a potential conflict of interest.
